# Das Deutsche Zentrum für Psychische Gesundheit: Innovative Translationsforschung zur Förderung von Prävention, zielgerichteter Intervention und Resilienz

**DOI:** 10.1007/s00115-024-01632-6

**Published:** 2024-03-15

**Authors:** Melissa G. Halil, Irina Baskow, Malte F. Zimdahl, Silke Lipinski, Rüdiger Hannig, Peter Falkai, Andreas J. Fallgatter, Silvia Schneider, Martin Walter, Andreas Meyer-Lindenberg, Andreas Heinz

**Affiliations:** 1 Deutsches Zentrum für Psychische Gesundheit (DZPG), Standort Berlin-Potsdam, Berlin, Deutschland; 2 Klinik für Psychiatrie und Psychotherapie, Charité Universitätsmedizin Berlin, Charité Platz 1, 10117 Berlin, Deutschland; 3 Deutsches Zentrum für Psychische Gesundheit (DZPG), Standort Mannheim-Heidelberg-Ulm, Heidelberg, Deutschland; 4 Klinik für Psychiatrie und Psychotherapie, Zentralinstitut für Seelische Gesundheit, Mannheim, Deutschland; 5 Aspies e. V. – Menschen im Autismusspektrum, Berlin, Deutschland; 6 Klinische Psychologie Sozialer Interaktion, Humboldt-Universität zu Berlin, Berlin, Deutschland; 7 Bundesverband der Angehörigen psychisch erkrankter Menschen e. V., Bonn, Deutschland; 8 Deutsches Zentrum für Psychische Gesundheit (DZPG), Standort München-Augsburg, München, Deutschland; 9 Klinik und Poliklinik für Psychiatrie und Psychotherapie, LMU Klinikum, LMU München, München, Deutschland; 10 Max-Planck-Institut für Psychiatrie, München, Deutschland; 11 Deutsches Zentrum für Psychische Gesundheit (DZPG), Standort Tübingen, Tübingen, Deutschland; 12 Abteilung für Psychiatrie und Psychotherapie, Universitätsklinikum Tübingen, Tübingen, Deutschland; 13 Deutsches Zentrum für Psychische Gesundheit (DZPG), Standort Bochum-Marburg, Bochum, Deutschland; 14 Klinische Kinder- und Jugendpsychologie, Forschungs- und Behandlungszentrum für psychische Gesundheit (FBZ), Ruhr-Universität Bochum, Bochum, Deutschland; 15 Deutsches Zentrum für Psychische Gesundheit (DZPG), Standort Halle-Jena-Magdeburg, Halle, Deutschland; 16 Klinik für Psychiatrie und Psychotherapie, Universitätsklinikum Jena, Jena, Deutschland; 17 Deutsche Gesellschaft für Psychiatrie und Psychotherapie, Psychosomatik und Nervenheilkunde e. V., Berlin, Deutschland; 18 Psychiatrische Universitätsklinik der Charité im St. Hedwig Krankenhaus, Berlin, Deutschland

**Keywords:** Psychische Erkrankungen, Stigmatisierung, Translation, Früherkennung, Partizipation, Mental disorders, Stigmatization, Translation, Early recognition, Participation

## Abstract

**Hintergrund:**

Aufgrund der hohen Krankheitslast, des frühen Beginns und der oft langfristigen Verläufe zählen psychische Erkrankungen zu den Volkskrankheiten mit wachsender Bedeutung. Das Deutsche Zentrum für Psychische Gesundheit (DZPG) wurde gegründet, um Forschungsbedingungen zu verbessern und versorgungsrelevante Ergebnisse schneller in die Praxis zu bringen.

**Ziel der Arbeit (Fragestellung):**

Das DZPG hat das Ziel, die psychische Gesundheitsversorgung in Deutschland zu optimieren, modifizierbare, gesellschaftliche Ursachen zu beeinflussen und Best-Practice-Modelle zur Versorgung vulnerabler Gruppen zu entwickeln. Es soll die psychische Gesundheit und Resilienz fördern, die Stigmatisierung psychischer Erkrankungen bekämpfen und dazu beitragen, die Behandlung dieser in allen Altersgruppen zu verbessern.

**Material und Methoden:**

Das DZPG nutzt ein translationales Forschungsprogramm, das die Übersetzung von Ergebnissen der Grundlagenforschung in die Klinik und deren breite Anwendung beschleunigt. Es werden Universitätsklinika und -ambulanzen, andere universitäre Fachbereiche und außeruniversitäre Forschungseinrichtungen eingebunden, um eine gemeinsam abgestimmte Infrastruktur für beschleunigte Translation und Innovation zu entwickeln.

**Forschungsschwerpunkte:**

Die Forschungsbereiche adressieren 1) die Interaktion psychischer und somatischer Risiko- und Resilienzfaktoren und Erkrankungen über die Lebensspanne, 2) die Beeinflussung relevanter modifizierbarer Umweltfaktoren und 3) darauf aufbauend die personalisierte Prävention und Intervention.

**Schlussfolgerungen:**

Das DZPG verfolgt das Ziel, innovative präventive und therapeutische Werkzeuge zu entwickeln, die eine verbesserte Versorgung psychisch erkrankter Menschen ermöglichen. Es beinhaltet eine umfassende Integration von Erfahrungsexpert:innen auf allen Entscheidungsebenen und trialogisch-partizipativ in allen Forschungsprojekten.

## Abstract (en)

**Background:**

Due to the high disease burden, the early onset and often long-term trajectories mental disorders are among the most widespread diseases with growing significance. The German Center for Mental Health (DZPG) was established to enhance research conditions and expedite the translation of clinically relevant findings into practice.

**Objective:**

The aim of the DZPG is to optimize mental healthcare in Germany, influence modifiable social causes and to develop best practice models of care for vulnerable groups. It seeks to promote mental health and resilience, combat the stigmatization associated with mental disorders, and contribute to the enhancement of treatment across all age groups.

**Material and methods:**

The DZPG employs a translational research program that accelerates the translation of basic research findings into clinical studies and general practice. University hospitals and outpatient departments, other university disciplines, and extramural research institutions are working together to establish a collaboratively coordinated infrastructure for accelerated translation and innovation.

**Research priorities:**

The research areas encompass 1) the interaction of somatic and mental risk and resilience factors and disorders across the lifespan, 2) influencing relevant modifiable environmental factors and 3) based on this personalized prevention and intervention.

**Conclusion:**

The DZPG aims to develop innovative preventive and therapeutic tools that enable an improvement in care for individuals with mental disorders. It involves a comprehensive integration of experts with experience at all levels of decision-making and employs trilogue and participatory approaches in all research projects.

Psychische Erkrankungen gehören aufgrund ihres häufigen Auftretens, ihres oft frühen Beginns und ihrer häufig langfristigen Verläufe zu den wichtigsten Volkskrankheiten. Um eine zielgerichtete und langfristige translationale Forschung im Zusammenspiel verschiedener Disziplinen (wie Mediziner:innen, Psycholog:innen, weitere Forschende, Betroffene) zu ermöglichen, wurde das Deutsche Zentrum für Psychische Gesundheit (DZPG) durch die Bundesregierung ausgeschrieben. Ziel ist es, Forschungsbedingungen zu optimieren, die Translation von Forschungsergebnissen aus der Grundlagenforschung zu beschleunigen, um so die Behandlung psychischer Erkrankungen langfristig spürbar zu verbessern.

## Hintergrund

**Figure 1:**
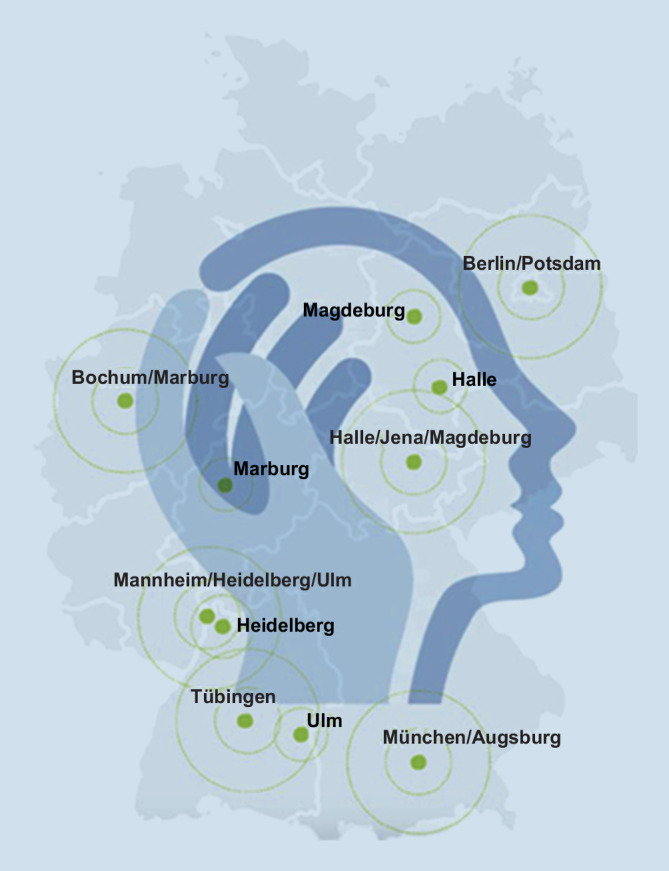

Forschung für eine verbesserte Prävention und Intervention bei psychischen Erkrankungen auf Ebene der Individuen, Familien und der Gesellschaft bildet den Grundstein für das Deutsche Zentrum für Psychische Gesundheit. Nach einer ersten Ausschreibungsphase wurden 6 Standorte (Berlin/Potsdam, Bochum/Marburg, Halle/Jena/Magdeburg, Mannheim/Heidelberg/Ulm, München/Augsburg, Tübingen) von einem internationalen Gremium in einem kompetitiven Prozess selektiert (Abb. 1). Diese ausgewählten Standorte erarbeiteten in einem zweiten Schritt ein gemeinsames Konzept mit dem Ziel, optimale Forschungsbedingungen zu schaffen und die Translation der Forschung in die klinische Anwendung zu beschleunigen.

Das DZPG strebt unter anderem Lösungen für gesellschaftliche Ungleichheiten in der psychischen Gesundheitsversorgung in städtischen und ländlichen Lebensräumen an und berücksichtigt vulnerable Gruppen, die besonders von psychischen Störungen betroffen sind, wie Erwerbslose und Menschen mit Migrationshintergrund. Das Zentrum setzt ein translationales Forschungsprogramm um, das die psychische Gesundheit und Resilienz fördert, das Bewusstsein für psychische Störungen schärft und die Prävention und Behandlung psychischer Erkrankungen in allen Altersgruppen verbessern soll. Dabei werden wichtige Dimensionen dieser Belastung, wie die Stigmatisierung, die erhöhte Sterblichkeit, das Zusammenspiel psychischer und körperlicher Erkrankungen sowie schlecht behandelte körperliche Folgeerscheinungen psychischer Erkrankungen und die Suizidalität, berücksichtigt.

Die beteiligten Einrichtungen der 6 Standorte des DZPG (Abb. 1) umfassen 42 Abteilungen an Universitätsklinika, 139 verbundene Lehrkrankenhäuser und versorgen jährlich insgesamt 48.560 stationäre und 224.000 ambulante Patient:innen. Die Ressourcen des DZPG werden in diesem Netzwerk auch genutzt, um Infrastrukturen zu entwickeln, die die Beschleunigung von Translation und therapeutischer Innovation ermöglichen. Hierzu gehören die Harmonisierung der Rekrutierung und Erfassung von Patient:innen, die Standardisierung und Vereinheitlichung des Datenmanagements, die Herstellung von Daten- und analytischer Interoperabilität sowie die Bereitstellung synergistischer Dienste und Plattformen für die Koordination von Forschungs‑, Bildungs- und Kommunikationsaktivitäten, die über die 6 Standorte des DZPG hinausgehen [1]. Von Anfang an hat das DZPG Patient:innen und Nutzer:innen von Dienstleistungen sowie ihre Angehörigen auf allen Ebenen des Netzwerks kontinuierlich einbezogen und ihre Expertise und Erfahrungen mit der Interdisziplinarität und Reichhaltigkeit von Ansätzen aus Grundlagen‑, Translations- und Implementierungswissenschaft zusammengebracht. Die Auswirkungen sozialer und anderer veränderbarer Umweltfaktoren auf das Auftreten und den Verlauf psychischer Störungen sowie die Nutzung von Wissen über Diversität zur personalisierten digitalen und persönlichen Prävention, Früherkennung und Behandlung stehen dabei im Vordergrund der Forschung.

Die Hauptziele des DZPG umfassen die forschungsgeleitete Prävention psychischer Erkrankungen in ihren frühestmöglichen Phasen, die Bekämpfung von Stigmatisierung, sozialer Ausgrenzung und Diskriminierung sowie das Verständnis und die Nutzung sozialer Prozesse und salutogener Faktoren, die der Genesung zugrunde liegen. Ein weiterer Fokus liegt auf der Stärkung von Gemeinschaften zur Inklusion von Menschen mit psychischen Erkrankungen. Innovative Ansätze zur Prävention und Intervention sollen eine höhere Widerstandsfähigkeit und bessere Behandlungsergebnisse ermöglichen. Für Patient:innen mit etablierten psychischen Erkrankungen entwickelt das DZPG Werkzeuge zur Stärkung ihrer Genesungsfähigkeit, einschließlich der sozialen und beruflichen Funktionsfähigkeit, wobei die jeweiligen Lebenswelten berücksichtigt werden. Ein weiteres Ziel des DZPG besteht darin, körperliche Erkrankungen zu adressieren, an denen Personen mit psychischen Erkrankungen früher versterben als Personen ohne psychische Gesundheitsprobleme [2]. Dazu sollen modifizierbare Umweltfaktoren adressiert werden, die für die Ätiologie, Manifestation, Prävention und personalisierte Therapie psychischer und damit verbundener körperlicher Erkrankungen von Relevanz sind.

Menschliche Diversität soll für Prävention und Intervention auf allen Ebenen berücksichtigt werden. Sie wird durch genetische Variabilität, soziale Unterschiede in Bezug auf Geschlecht, Alter, Bildung, schädliche/gesundheitsfördernde Erfahrungen und Verhaltensweisen, Einkommen und Status, Obdachlosigkeit sowie individuelle Unterschiede im Zusammenspiel somatischer und psychischer Erkrankungen über die Lebensspanne bestimmt [3–5]. Das DZPG vereint dafür Expertise in drei miteinander verbundenen Forschungsbereichen:

1. Entsprechend dem Ansatz der Research Domain Criteria (RDoC [6]) erforscht das DZPG Mechanismen, die den Lebensverlauf der psychischen Gesundheit und deren Beziehung zur physischen Gesundheit durch prospektive, multimodale, tiefgreifende Phänotypisierungs- und (epi)genetische Studien untersuchen [7–9].
2. In epidemiologisch repräsentativen Stichproben und prospektiven Kohorten untersucht das DZPG die Auswirkungen sozialer und anderer veränderbarer Umweltfaktoren auf die psychische und physische Gesundheit [4, 10, 11].
3. Das DZPG wendet Wissen über individuelle und soziale Diversität in den Verläufen psychischer und somatischer Erkrankungen an, um mechanismenbasierte, zielgerichtete digitale und persönliche Prävention, Früherkennung und Behandlung psychischer Erkrankungen umzusetzen. Dabei soll die Translation von Grundlagenforschung in klinische Studien und die allgemeine Praxis beschleunigt werden, und klinische Erfahrungen sollen auf die Grundlagenforschung zurückwirken (Back-Translation; [5, 12, 13]).

**Figure 2:**
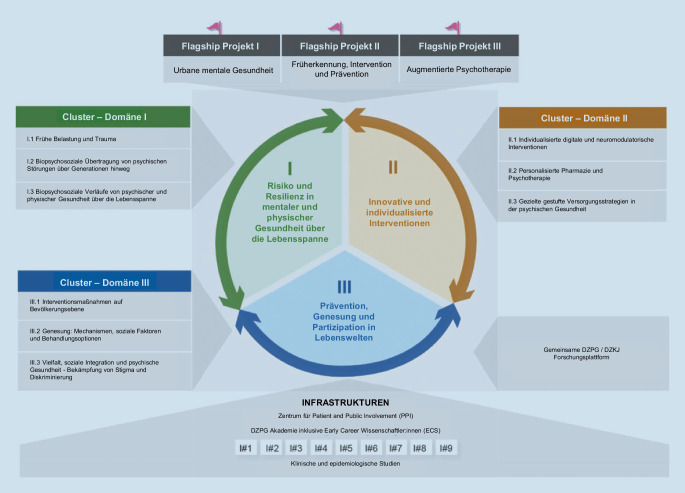

Entsprechend hat das DZPG den Translationszyklus in drei Domänen unterteilt, die „Risiko und Resilienz“, „Lebenswelten“ und „Interventionen“ abdecken [14]. Jede Domäne ist wiederum in drei Cluster unterteilt, die kritische Themen für die translationale Forschung umfassen (Abb. 2).

## Die drei Forschungsdomänen des DZPG

### Domäne I

Ein Hauptfokus der Domäne I liegt auf „früher Belastung und Trauma“ als Hauptrisikofaktoren für psychische und körperliche Erkrankungen über die Lebensspanne mit ungünstigen Langzeitfolgen. Dies geschieht beispielsweise über Stress, immunologische und lernmechanistische Prozesse. Die longitudinale Komponente der „transgenerationalen Übertragung“ ist ein wichtiger Aspekt, der die ggf. lebenslange schädliche Wirkung von Traumata berücksichtigt. Omics-, kognitive, Verhaltens- und Sozialmethoden sowie innovative Modellsysteme und prädiktive Werkzeuge sollen es ermöglichen, die „biopsychosozialen Verläufe“ psychischer Erkrankungen und die Interaktion psychischer und somatischer Erkrankungen auf individueller und gemeinschaftlicher Ebene abzuschätzen [15]. Das Ziel ist die Identifikation salutogener und pathogener Verläufe und ihrer Vorhersagefaktoren, wie sie beispielsweise durch langfristige Ergebnisse oder das Ansprechen auf Behandlung definiert werden.

Domäne I erweitert das Verständnis der Ursachen und Verläufe psychischer Erkrankungen, komorbider körperlicher Erkrankungen und salutogener Prozesse, die Resilienz gegenüber psychischen Erkrankungen vermitteln. Die Domäne I umfasst zwei der Hauptrisikofaktoren für psychische Erkrankungen (frühe Belastung und psychische Gesundheit der Familie) und deren dynamische Wechselwirkung mit biopsychosozialen Entwicklungsprozessen über die Lebensspanne.

### Domäne II

Domäne II konzentriert sich auf die Prüfung und Verbesserung psychosozialer, psychotherapeutischer, pharmakologischer sowie hirnstimulations- und neurofeedbackbasierter Interventionen sowie deren Kombinationen in transdiagnostischen Patient:innengruppen. Das DZPG strebt an, Behandlungsoptionen neu zu positionieren, zu verbessern, zu personalisieren und sie in die routinemäßige klinische Praxis sowie die gemeindebasierte Gesundheitsversorgung zu implementieren. Die Implementierung umfasst die schnelle Umsetzung neuer therapeutischer Erkenntnisse durch digitalisierte und mehrsprachige Leitlinien sowie die Förderung der psychischen Gesundheitskompetenz durch eine nationale Verbreitungsstrategie.

Domäne II entwickelt neue Interventionen, die auf einem tieferen Verständnis neurobiologischer, psychologischer und sozioökologischer Mechanismen beruhen, und kombiniert bestehende und innovative Interventionen. Skalierbare Lösungen und gestufte Versorgungsalgorithmen sollen den Nutzen für die Patient:innen optimieren.

### Domäne III

Domäne III untersucht Prävention, Partizipation und Genesung in relevanten Lebenswelten. Das übergeordnete Ziel von Domäne III besteht darin, Grundlagenforschung und klinische Studienergebnisse an die vielfältigen Umgebungen anzupassen, in denen Patient:innen, Angehörige und Fachleute leben und interagieren, gerade auch angesichts des Klimawandels. Die Komplexität der Lebenswelten wird durch multimodale Tiefencharakterisierung in verknüpften und harmonisierten Kohortenstudien erfasst und bildet die Grundlage für bevölkerungsbasierte Interventionen, die Prävention und Genesung fördern. Stigmatisierung und soziale Ausgrenzung sind so wichtige Probleme, dass ihnen ein separates Forschungscluster gewidmet wird.

Domäne III stellt einen entscheidenden Bereich für partizipative Forschung zu salutogenen Prozessen dar.

### Beschleunigung der Translation und Back-Translation

Die Beschleunigung der Translation und Back-Translation soll nun einerseits erfolgen, indem individuelle Verläufe (Domäne I) in ihren jeweiligen Lebenswelten (Domäne III) untersucht werden, um darauf aufbauend gezielt zu intervenieren (Domäne II). Translation ist jedoch auch in anderer Richtung denkbar, nämlich von individuellen Verläufen (Domäne I) über Interventionen (Domäne II) in die leitliniengerechte Implementierung solcher therapeutischen und präventiven Ansätze (Domäne III). Die Beschleunigung der Translation soll hierbei vor allem durch drei Faktoren erfolgen:

1. eine integrierte Infrastruktur, die sämtliche Studiendaten über alle Standorte hinweg vergleichbar macht, in dem ein gemeinsamer Basisdatensatz sowohl klinisch wie mittels Ecological Momentary Assessments (App-gestützte Erfassung von beispielsweise Stress- und Risikofaktoren, psychischer Befindlichkeit und Drogenkonsum) erfolgen soll [1];
2. durch Translation und Back-Translation von Grundlagenforschung in klinische Studien und klinischer und epidemiologischer Daten in gezielte Fragen an die Grundlagenforschung; dies geschieht auch im Besonderen durch die Tatsache, dass Projektleitende sowohl Forschende als auch praktizierende Kliniker:innen sind und oft auch diese beiden Erfahrungsbereiche als Scientist-Practitioners gemeinsam vertreten;
3. die Einbeziehung von Angehörigen und Betroffenen auf allen Entscheidungsebenen und insbesondere bei allen Forschungsprojekten im Sinne der Partizipation soll dafür sorgen, dass die Forschungsfragen praxisnah und mit hoher Relevanz für Prävention und klinischer Versorgung gestellt werden.

## Das Flagship-Programm

Innerhalb der drei Forschungsdomänen beschäftigt sich das Flagship-Programm des DZPG mit drei ausgewählten Schwerpunkten:

- urbane psychische Gesundheit,
- Früherkennung, Intervention und Prävention sowie
- augmentierte Psychotherapie.

Diese Projekte dienen als zentrale Initiativen, um Forschungsaktivitäten an allen DZPG-Partnerstandorten zu integrieren und innovative Lösungen für bislang ungedeckte psychische Gesundheitsbedürfnisse zu fördern.

### Urbane psychische Gesundheit

Im ersten Flagship-Projekt liegt der Fokus auf der psychischen Gesundheit in urbanen Gebieten („urban mental health“ [4, 16]). Stadtleben stellt einen kausalen Risikofaktor für psychische Störungen dar, insbesondere für sozial benachteiligte Minderheiten [17, 18]. Das DZPG strebt eine Verbesserung der psychischen Gesundheit der Lebenswelten an und beschleunigt den Zugang zu psychosozialen Diensten durch niedrigschwellige, evidenzbasierte Interventionen. Es wird ein nationales Netzwerk genutzt, um städtische und ländliche Regionen mittels eines harmonisierten Erhebungskonzepts zu untersuchen und eine umfassende Charakterisierung der Lebenswelten in ganz Deutschland zu schaffen. Dies ermöglicht nicht nur eine Verbesserung der psychischen Gesundheit in urbanen Gebieten, sondern auch Erkenntnisse über Risiko- und Resilienzprozesse in verschiedenen Lebensräumen.

### Früherkennung, Intervention und Prävention

In der zweijährigen Aufbauphase des DZPG liegt ein besonderer Schwerpunkt auf Flagship 2. Dieses widmet sich der Früherkennung, Intervention und Prävention psychischer Störungen („early recognition, intervention and prevention“). Ziel ist es, breit zugängliche, evidenzbasierte Präventions- und Frühinterventionsinstrumente zu entwickeln und zu etablieren, um die Versorgungspraxis signifikant zu verbessern. Hierfür werden computergestützte Prognose-Tools entwickelt, die individuelle und soziale Risikoverläufe quantifizieren, um optimale multimodale Interventionen für gefährdete Personen auszuwählen. Personalisierte Interventionen sollen das Erkrankungs- und Chronizitätsrisiko senken. Die Entwicklung abgestufter, individuell anpassbarer, multimodaler Behandlungskonzepte ist von zentraler Bedeutung und erfordert die Zusammenarbeit von Kliniker:innen, Patient:innen, Angehörigen im Zusammenspiel mit prädiktiven auf künstliche Intelligenz (KI-)basierten Verfahren [19, 20].

### Augmentierte Psychotherapie

Das dritte Flagship-Projekt widmet sich der augmentierten Psychotherapie („enhanced psychotherapy“). Hier soll Psychotherapie nicht nur durch Medikamente, sondern auch beispielsweise durch Hirnstimulationsverfahren, Neurofeedback, virtuelle Realität und digitale Interventionen verstärkt werden. Obwohl psychotherapeutische Interventionen Fortschritte gemacht haben, sind ihre Mechanismen noch nicht vollständig verstanden. Der Ansatz der „enhanced psychotherapy“ basiert auf einem mechanistischen Verständnis und zielt darauf ab, langfristige und nachhaltige Verbesserungen der Behandlungsergebnisse zu erzielen. Durch die Kombination neuer Erkenntnisse und die optimierten Bedingungen des DZPG wird ein großer translationaler Schritt nach vorne gemacht. Die Integration von Lernen, Gedächtnis und neuronaler Plastizität als Grundlage psychotherapeutischer Behandlungen soll die Ergebnisse für Patient:innen verbessern [21, 22].

## Erfahrungsexpertise und Partizipation

Das Forschungsprogramm des DZPG zeichnet sich durch eine Besonderheit aus: Es integriert systematisch Erfahrungsexpert:innen – Betroffene und Angehörige – in sämtliche Phasen der Forschungsgestaltung, was bisher in diesem Fachgebiet eher unüblich war^1^. Das Gesamtkonzept des DZPG enthält systematische Partizipationsstrukturen auf allen Entscheidungsebenen. So haben Betroffene und Angehörige in der Gestaltungsphase des gemeinsamen Antrags je eine Stimme, die 6 Standorte jeweils auch, d. h. 25 % der entscheidungsrelevanten Stimmenanteile werden durch Betroffene und Angehörige genutzt. So wurde eine zentrumsübergreifende Strategie entwickelt, um die partizipatorischen Strukturen und Abläufe innerhalb des DZPG zu sichern [23].

Bereits im Sommer 2021, noch vor der eigentlichen Konzeptentwicklungsphase, wurde ein Trialogischer Zentrumsrat ins Leben gerufen. Dieser Rat setzt sich aus Betroffenen, Angehörigen und Forschenden der verschiedenen DZPG-Standorte zusammen. Dadurch wird gewährleistet, dass die Einbindung von Erfahrungsexpert:innen bereits bei der Gestaltung der zukünftigen Strukturen und inhaltlichen Schwerpunkte erfolgt. Der Zentrumsrat hat die wichtige Aufgabe, Themen wie „Peer-Support, Entstigmatisierung, Salutogenese, Umgang mit Angehörigen und partizipatives Forschen in der klinischen Psychologie und Psychiatrie“ in die Forschungsagenda des DZPG zu integrieren. Um nachhaltige Strukturen und Möglichkeiten der Mitbestimmung im DZPG zu schaffen, wurde eine Abteilung für „Patient and Public Involvement“ entwickelt. An den einzelnen Standorten werden zudem lokale Räte etabliert, die über Entscheidungsbefugnisse verfügen. Diese lokalen Räte sind an der partizipativen Gestaltung der Forschungsprojekte systematisch beteiligt [24].

## Die Governance-Struktur

**Figure 3:**
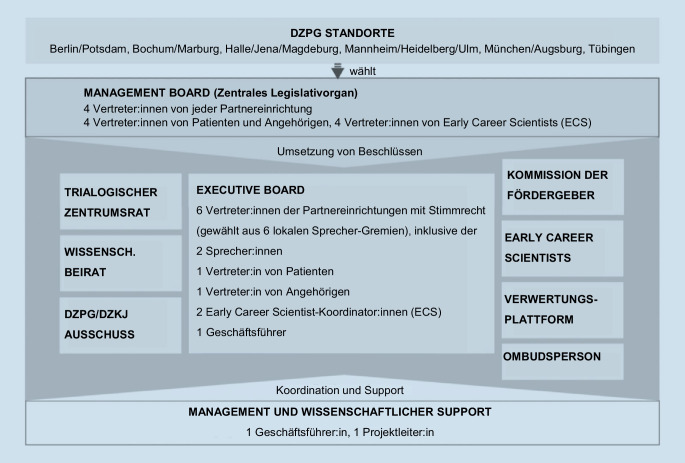

Die administrative Governance-Struktur des DZPG besteht aus Lenkungsgremien an den einzelnen Standorten, die jeweils 4 Vertreter:innen (einschließlich Sprecher:innen und stellvertretenden Sprecher:innen) wählen, dem Trialogischen Zentrumsrat der Angehörigen und Betroffenen sowie der Organisation der Early Career Scientists (ECS). Diese Vertreter:innen bilden gemeinsam das standortübergreifende Management Board des DZPG. Das Management Board tagt monatlich und fungiert als zentrales Austauschgremium für die wissenschaftliche Strategie des Zentrums, einschließlich der Bereiche und Cluster. Es wählt gemeinsame Forschungsprojekte aus und koordiniert die Zusammenarbeit zwischen den Partnerstandorten in Bezug auf Projekte und Infrastrukturen (Abb. 3).

Das Management Board ist auch zuständig für die Ausrichtung der interdisziplinären Zusammenarbeit mit anderen Deutschen Zentren der Gesundheitsforschung, insbesondere dem Deutschen Zentrum für Kinder- und Jugendgesundheit (DZKJ), mit dem eine gemeinsame Plattform etabliert wird, sowie mit weiteren Partnern außerhalb der DZPG-Community. Es ernennt auch die Mitglieder des wissenschaftlichen Beirats und die Geschäftsführung des Zentrums. Jeder Partnerstandort im Management Board hat eine Stimme (insgesamt 6 Stimmen), die Vertreter:innen der Early Career Scientists (ECS^2^) haben eine Stimme, und die Vertreter:innen von Betroffenen und Angehörigen haben zwei Stimmen^3^ – insgesamt 9 Stimmen. Wissenschaftliche Entscheidungen müssen von der Mehrheit der Standorte unterstützt werden.

Das Executive Board wird von den Sprecher:innen aller Partnerstandorte, den Early Career Scientists sowie dem zentralen trialogischen Board benannt. Das Executive Board kontrolliert die Finanzen und berichtet vierteljährlich dem Management Board über die umgesetzten Maßnahmen. Die Executive-Board-Mitglieder werden für 4 Jahre ernannt. Die beiden Sprecher:innen des DZPG werden für 2 Jahre gewählt und stehen in engem Kontakt mit den Fördermittelgebenden (Bundesministerium für Bildung und Forschung/DLR-PT). In den ersten beiden Jahren übernehmen die Partnerstandorte Berlin (Andreas Heinz) und Mannheim (Andreas Meyer-Lindenberg) gleichberechtigt die Leitung, gefolgt von Bochum und München in den nächsten 2 Jahren und anschließend Jena und Tübingen in den darauffolgenden 2 Jahren, bevor der Zyklus erneut beginnt.

## Fazit für die Praxis

- Das übergreifende Ziel des Deutschen Zentrums für Psychische Gesundheit (DZPG) ist die Optimierung von Forschungsbedingungen zur Beschleunigung der Translation und Back-Translation, um psychische Erkrankungen besser zu verstehen, versorgungsrelevante Forschungsergebnisse schneller in die Praxis zu bringen und damit die Belastungen durch psychische Erkrankungen spürbar zu reduzieren.
- Hauptbereiche des DZPG sind die Mechanismen, die den Lebensverlauf psychischer und physischer Gesundheit beeinflussen, die Auswirkungen sozialer und anderer veränderbarer Umweltfaktoren auf das Auftreten und den Verlauf psychischer Störungen und die Nutzung von Wissen über Diversität zur personalisierten digitalen und persönlichen Prävention, Früherkennung und Behandlung.
- Eine Besonderheit des DZPG ist die enge und systematische Einbeziehung der Betroffenen und Angehörigen in allen Phasen der Planung, Durchführung und Umsetzung der Forschungsgestaltung und Ergebnisse.

## Footnotes

1 Für weitere Informationen zu Erfahrungsexpertise und Partizipation, siehe Artikel „Patient and Public Involvenment am Deutschen Zentrum für Psychische Gesundheit“ in dieser Ausgabe von *Der Nervanarzt*.

2 Für weitere Informationen zu Early Career Scientists (ECS) siehe Artikel „Strukturen, Ziele und Bedürfnisse der Early Career Scientists am Deutschen Zentrum für Psychische Gesundheit“ in dieser Ausgabe von *Der Nervenarzt*.

3 Für weitere Informationen zu Erfahrungsexpert:innen siehe Artikel „Patient and Public Involvenment am Deutschen Zentrum für Psychische Gesundheit“ in dieser Ausgabe von* Der Nervenarzt*.
